# Correlated evolution of LTR retrotransposons and genome size in the genus *eleocharis*

**DOI:** 10.1186/1471-2229-10-265

**Published:** 2010-11-30

**Authors:** František Zedek, Jakub Šmerda, Petr Šmarda, Petr Bureš

**Affiliations:** 1 Department of Botany and Zoology, Masaryk University, Kotlářská 2, 61137 Brno, Czech Republic

## Abstract

**Background:**

Transposable elements (TEs) are considered to be an important source of genome size variation and genetic and phenotypic plasticity in eukaryotes. Most of our knowledge about TEs comes from large genomic projects and studies focused on model organisms. However, TE dynamics among related taxa from natural populations and the role of TEs at the species or supra-species level, where genome size and karyotype evolution are modulated in concert with polyploidy and chromosomal rearrangements, remain poorly understood. We focused on the holokinetic genus *Eleocharis *(*Cyperaceae*), which displays large variation in genome size and the occurrence of polyploidy and agmatoploidy/symploidy. We analyzed and quantified the long terminal repeat (LTR) retrotransposons Ty1-*copia *and Ty3-*gypsy *in relation to changes in both genome size and karyotype in *Eleocharis*. We also examined how this relationship is reflected in the phylogeny of *Eleocharis*.

**Results:**

Using flow cytometry, we measured the genome sizes of members of the genus *Eleocharis *(Cyperaceae). We found positive correlation between the independent phylogenetic contrasts of genome size and chromosome number in *Eleocharis*. We analyzed PCR-amplified sequences of various *reverse transcriptases *of the LTR retrotransposons Ty1-*copia *and Ty3-*gypsy *(762 sequences in total). Using real-time PCR and dot blot approaches, we quantified the densities of Ty1-*copia *and Ty3-*gypsy *within the genomes of the analyzed species. We detected an increasing density of Ty1-*copia *elements in evolutionarily younger *Eleocharis *species and found a positive correlation between Ty1-*copia *densities and C_/n_-values (an alternative measure of monoploid genome size) in the genus phylogeny. In addition, our analysis of Ty1-*copia *sequences identified a novel retrotransposon family named Helos1, which is responsible for the increasing density of Ty1-*copia*. The transition:transversion ratio of Helos1 sequences suggests that Helos1 recently transposed in later-diverging *Eleocharis *species.

**Conclusions:**

Using several different approaches, we were able to distinguish between the roles of LTR retrotransposons, polyploidy and agmatoploidy/symploidy in shaping *Eleocharis *genomes and karyotypes. Our results confirm the occurrence of both polyploidy and agmatoploidy/symploidy in *Eleocharis*. Additionally, we introduce a new player in the process of genome evolution in holokinetic plants: LTR retrotransposons.

## Background

Transposable elements (TEs) have been found in all eukaryotic genomes investigated [1]. The most numerous group of TEs are long terminal repeat (LTR) retrotransposons, namely, the Ty1-*copia *and Ty3-*gypsy *superfamilies, which have become extremely abundant in plant genomes over time [2,3]. These LTR retrotransposons copy themselves through a process of replicative transposition and are thus able to increase the genome size of the host [4,5]. Retrotransposon insertion in close proximity to or directly into genes greatly affects gene expression and function [6-8]. Recombination between retrotransposons can cause large chromosomal rearrangements, including inversions, translocations, deletions and duplications [9,10]. Hence, retrotransposons are an important source of genetic and phenotypic diversity in plants [2,11,12].

Potentially deleterious processes such as retrotransposition must be moderated so as not to have damaging effects on the host. Therefore, LTR retrotransposons are epigenetically silenced (i.e., methylated) in plant genomes [13] and have been under strong purifying selection pressure throughout much of evolutionary time [14-16]. LTR retrotransposons are also actively removed from the genomes of host species, although this process is much slower than retrotransposon proliferation [10]. Retrotransposons also respond to various stress stimuli, such as environmental changes and polyploid formation [12,17-20]. Usually, only a limited number of LTR retrotransposon families successfully populate the genome. For instance, only three families are responsible for doubling the genome size of *Oryza australiensis *within the last three million years [21], and nearly 10% of the barley genome is occupied by BARE-1 *copia*-like elements [22]. In addition, BARE-1 proliferation has been proposed as an evolutionary driving force in natural barley populations [23,24].

Most studies of TE behavior have been restricted to large genomic projects, which tend to be focused on model organisms. Little is known about TE dynamics in natural populations or among related taxa, and even less is understood about the role of TEs at the species or supra-species level, where they can modulate genome size and karyotype evolution in concert with polyploidy and chromosomal rearrangements.

An appropriate model group for evaluating the evolutionary dynamics of retrotransposons on the microevolutionary scale should exhibit reasonable monoploid genome size variation within a particular taxon (section, subgenus, genus or family). Among angiosperm species in which both the ploidy level (or chromosome number) and genome size are known [25], one of the largest contrasts is reported in the genus *Eleocharis *(*Cyperaceae*). *E. acicularis *(2n = 20; 2C = 0.50 pg) and *E. palustris *(2n = 16; 2C = 11.05 pg) differ almost 22-fold in somatic DNA quantity at approximately the same ploidy level. This contrast is noticeable when compared with the majority of species in the family *Cyperaceae*, which exhibit smaller genomes.

The genus *Eleocharis *comprises more than 250 annual or perennial species of marsh and wetland habitats [26]. The species samples included in our study were selected to represent the genus phylogeny (all four subgenera: *Limnochloa*, *Zinserlingia*, *Scirpidium *and *Eleocharis*). Here we analyzed the evolutionary dynamics of the LTR retrotransposons Ty1-*copia *and Ty3-*gypsy *in relation to genome size and chromosome number (or size). In addition, we analyzed independent phylogenetic contrasts and signals [27] to determine how this relationship is reflected in the phylogeny of *Eleocharis*.

## Results and Discussion

### Phylogeny of analyzed species

**Figure 1:**
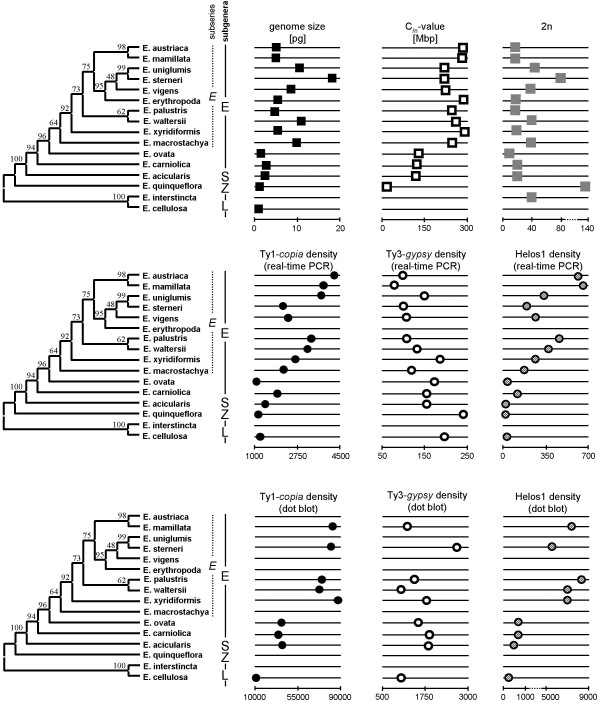
**Patterns of LTR retrotransposons in the phylogeny of *Eleocharis***. An unrooted neighbor-joining phylogenetic tree of analyzed *Eleocharis *species based on concatenated alignments of the *ppc *gene and ITS sequences is shown. Numbers above branches indicate bootstrap values. Subgenera of *Eleocharis sensu *González-Elizondo and Peterson [67]: E = *Eleocharis subgenus Eleocharis*, S = *Eleocharis subgenus Scirpirdium*, Z = *Eleocharis subgenus Zinserlingia*, L = *Eleocharis subgenus Limnochloa*. Subseries of *Eleocharis*: *E *= *Eleocharis subgenus Eleocharis subseries Eleocharis*. Densities (number of copies per pg of genomic DNA) of the *reverse transcriptases *of LTR retrotransposons estimated by both dot blot and real-time PCR are indicated by circles. Genome size, C**_/n_**-value and chromosome number (2n) are indicated by squares.

We reconstructed the phylogeny of the analyzed *Eleocharis *species. The neighbor-joining tree shown in Figure 1 is based on concatenated alignments of consensus ITS sequences and partial sequences of the *phosphoenolpyruvate carboxylase *(*ppc*) gene (see Methods and Additional file 1). The topology of the tree agrees with previously published phylogenies of *Eleocharis*, with the subgenus *Limnochloa *at the base of the tree, followed by the subgenera *Zinserlingia *and *Scirpidium*, and with the type subgenus (subgenus *Eleocharis*) as the evolutionarily youngest taxon [26,28,29].

### Correlated evolution of genome size and chromosome number in *Eleocharis*

Genome size was measured using flow cytometry (see Methods). For most of the analyzed species, genome size is presented here for the first time (Table 1). The somatic DNA quantities estimated for *E. acicularis *(Table 1) differ significantly from those reported in the Plant DNA C-values database (0.50 pg [25]). Therefore, we sampled seven different populations and detected no significant intraspecific variation (2C = 2.47; standard deviation = 0.03 pg; N = 7). *Eleocharis palustris *ssp. *waltersii *(2n = 38, 39) is widespread in Great Britain, whereas *E. palustris *ssp. *palustris *(2n = 16) is quite rare (see [30]). Thus, the value from the Plant DNA C-values database (2C = 11.05 pg, [25]) refers to the former rather than the latter, which is in accordance with our observations (Table 1).

**Table 1:** List of analyzed *Eleocharis *species and measured values

	2C [pg]	2n^1)^	2C/2n [Mbp]	LTR retrotransposon densities (real-time PCR)			LTR retrotransposon densities (dot blot)		
				
				Ty1-*copia*	Ty3-*gypsy*	Helos1	Ty1-*copia*	Ty3-*gypsy*	Helos1
*E. acicularis*	2.47^2)^	20	121	1507	158	4	36671	1859	686
*E. austriaca*	4.84	16	296	4290	101	622	n.a.	n.a.	n.a.
*E. carniolica*	2.58	20	126	1971	159	133	29843	2064	801
*E. cellulosa*	0.84	n.a.	n.a.	1236	193	10	9875	918	300
*E. eryhtropoda*	5.48	18	298	n.a.	n.a.	n.a.	n.a.	n.a.	n.a.
*E. interstincta*	n.a.	40/52	n.a.	n.a.	n.a.	n.a.	n.a.	n.a.	n.a.
*E. macrostachya*	9.80	38	253	2217	119	181	n.a.	n.a.	n.a.
*E. mamillata*	4.60	16	282	3928	89	677	85510	1279	7186
*E. ovata*	1.40	10	137	1007	174	19	35417	1517	816
*E. palustris*	4.20	16	257	3464	113	435	79966	1445	8132
*E. quinqueflora*	1.12	136	8	1112	241	2	n.a.	n.a.	n.a.
*E. sterneri*	18.00	74	238	2217	107	199	n.a.	n.a.	n.a.
*E. uniglumis*	10.20	46	217	3899	149	320	81196	2845	5634
*E. vigens*	8.74	36	238	2507	111	243	n.a.	n.a.	n.a.
*E. waltersii*	11.00	39	276	3319	131	386	70679	900	6735
*E. xyridiformis*	5.50	18	299	2739	183	291	92801	1792	6676
Significance of phylogenetic signal	-	*	**	*	*	**	**	-	**

1) Chromosome numbers were taken from previous studies ([68]: *E. palustris, E. waltersii*; [49]: E. sterneri because of sample identity; [69]: *E. quinqueflora *because of population identity; [35]: *E. interstincta*). For the remaining samples, chromosome numbers were counted by O. Rotreklová and P. Bureš (unpublished data).

2) An average of seven samples, each representing a different population (see Results and Discussion).

* p < 0.05; ** p < 0.01

Compared with other *Cyperaceae *and most other angiosperm genera, *Eleocharis *species vary greatly in genome size (Table 1[25]). The size of *Eleocharis *genomes (2C) range from 0.84 pg in *E. cellulosa *to 18 pg in *E. sterneri *(Table 1), which is an almost 22-fold difference.

We found that genome size (2C) is significantly correlated with chromosome number in the phylogeny of *Eleocharis *(2n, Figure 1; R = 0.918, p < 0.01, N = 12), suggesting that polyploidy plays an important role in generating genome size variation between *Eleocharis *species. This result is in strong contrast with the negative correlation found for the *Cyperaceae *genus *Carex *[31], in which polyploidy is considered to be very rare [32]. Such a negative correlation was also reported as a general pattern in the phylogenetic lineage comprising sister families *Juncaceae *and *Cyperaceae *[33]. The only exception to the observed trend is *E. quinqueflora*, which has a small genome (2C = 1.12 pg) and high chromosome number (2n = 136). It is likely that a similar deviation also exists in species of the subgenus *Limnochloa*, which are predicted to have small genomes based on the DNA content estimated for *E. cellulosa *(2C = 0.84 pg) and which have chromosome numbers ranging from 40 to 200 **[**34,35**]**. Nevertheless, the high chromosome numbers observed in the basal clades *Zinserlingia *and *Limnochloa *resulted rather from agmatoploidy (chromosomal fission) than polyploidy [35], similar to *Carex *(see discussion above). Thus, higher polyploids appear to belong to the evolutionary youngest subgenus of *Eleocharis*.

The contrast in genome size between basal and younger clades of the genus *Eleochris *becomes more apparent if chromosome size is taken into account (Table 1). It has been suggested that symploidy (chromosomal fusion) is responsible for this pattern [32]. However, in addition to symploidy or agmatoploidy, which are the widespread chromosomal rearrangements generally thought to be responsible for dysploidy across the holokinetic family *Cyperaceae *[33,34], transposable element proliferation should also be considered (see Background).

To distinguish between the roles of TEs and chromosomal rearrangements in chromosome size variation, we examined the relationship between TE density (copy number per pg of genomic DNA) and average chromosome size (C_/n_-value; [36] ). The C_/n_-value is a species-specific parameter calculated by dividing the amount of somatic DNA by the number of somatic chromosomes (2C/2n) (Table 1). If a change in C_/n _is only the result of a rearrangement, the TE density in the whole genome should remain unchanged. On the other hand, if TEs are active players, their density should mirror the changes in average chromosome size.

In the genus *Eleocharis*, the observed C_/n_-values increase toward evolutionary younger taxa and show a highly significant phylogenetic signal (Table 1 Figure 1). The small C_/n _in *E. quinqueflora *can be explained by agmatoploidy, whereas the increase in chromosome size in more recently diverged taxa (Figure 1; Table 1) could result either from fusion or from the amplification of transposable elements, most likely LTR retrotransposons.

To assess the contribution of LTR retrotransposons to this C_/n _divergence, we analyzed and quantified partial sequences from both LTR retrotransposon superfamilies. We used previously described primers to isolate the *reverse transcriptase *sequences of Ty1-*copia *and Ty3-*gypsy *[37-39] from all analyzed species. PCR, cloning and sequencing yielded 422 and 340 partial sequences of the *reverse transcriptase *sequences of Ty1-*copia *and Ty3-*gypsy*, respectively [GenBank: GU976288-GU977049; Additional files 2, 3 and 4]. BLAST comparison confirmed their homology to previously described *copia*-like or *gypsy*-like elements. To determine whether species with larger C_/n _values carry more retrotransposon copies, we quantified the *reverse transcriptase *sequences of Ty1-*copia *and Ty3-*gypsy *in each species using real-time PCR with the same pairs of primers (see Methods). Because the real-time PCR approach analyzed only a subset of retrotransposons, as defined by the specific primer pairs, dot blot quantification was also carried out in selected species to corroborate the real-time PCR results.

### Ty1-*copia *density correlates with C_/n_-value in the phylogeny of *Eleocharis*

As expected, the TE densities obtained from the dot blot quantification were higher (by one order of magnitude) than the densities estimated by quantitative PCR (Table 1). The estimates of both methods were positively correlated only for Ty1-*copia*. However, the differences in Ty3-*gypsy *density between analyzed species were very small and possibly below the resolution of both approaches, especially the dot blot analysis.

The calculated densities of Ty1-*copia *and Ty3-*gypsy *differed by one order of magnitude in favor of Ty1-*copia *(Table 1), suggesting that Ty1-*copia *is more successful in populating *Eleocharis *genomes than Ty3-*gypsy*. In addition, the density of Ty1-*copia *increases with evolutionarily younger taxa on the tree, whereas the density of Ty3-*gypsy *remains unchanged (dot blot) or slightly decreases (real-time PCR) (Figure 1). This pattern might be explained by the higher amplification rate of Ty1-*copia *(compared to Ty3-*gypsy*) in recently diverged species. If Ty3-*gypsy *remains inactive or nearly inactive while Ty1-*copia *proliferates, the density of Ty3-*gypsy *should be decreasing. A higher activity of Ty3-*gypsy *in basal taxa might be another reason for the resulting pattern. A phylogenetic analysis of Ty3-*gypsy reverse transcriptase *sequences revealed that 18 out of 28 (64%) sequences from *E. quinqueflora *formed a well-supported species-specific clade (Additional file 5). Species-specific and strongly supported (bootstrap values > 80%) clades of Ty3-*gypsy *with at least five sequences were also detected in *E. cellulosa, E. interstincta *and *E. ovata *(Additional file 5). The presence of species-specific clades suggests that these species underwent a burst of Ty3-*gypsy *amplification after diverging from their common ancestor. It is also possible that Ty3-*gypsy *is more efficiently removed from *Eleocharis *genomes than Ty1-*copia*. However, the preferential removal of an entire retrotransposon superfamily is unlikely.

The increased Ty1-*copia *density in *Eleocharis *is accompanied by an increase in C_/n _(Figure 1). The phylogenetic relationship between C_/n _and Ty1*-copia *density is significant (Table 2), implying that the larger C_/n_-values in recently diverged species resulted from an amplification of Ty1-*copia *elements. This correlation is particularly apparent in species from the subseries *Eleocharis*, in which the doubling of C_/n _is mirrored by a doubling of Ty1-*copia *densities as indicated by dot blot analysis (Figure 1; Table 1).

**Table 2:** Correlation between retrotransposon density and average chromosome size

	retrotransposon density (real-time PCR)					
	Ty1-*copia*		Ty3-*gypsy*		Helos1	
Spearman correlation^1^	n	R	n	R	n	R
**C_/n_-value**	13	0.770**	13	-0.505	13	0.835**
						
Independent contrasts^2^	n	R	n	R	n	R
**C_/n_-value**	11	0.510	11	-0.461	11	0.571*

	**retrotransposon density (dot blot)**					
	Ty1-*copia*		Ty3-*gypsy*		Helos1	

Spearman correlation^1^	n	R	n	R	n	R
**C_/n_-value**	8	0.833*	8	-0.548	8	0.786*
						
Independent contrasts^2^	n	R	n	R	n	R
**C_/n_-value**	7	0.918**	7	-0.353	7	0.914**

1) "n" indicates the number of correlated values

2) "n" indicates the number of independent phylogenetic contrasts

* p < 0.05; ** p < 0.01

**Figure 2:**
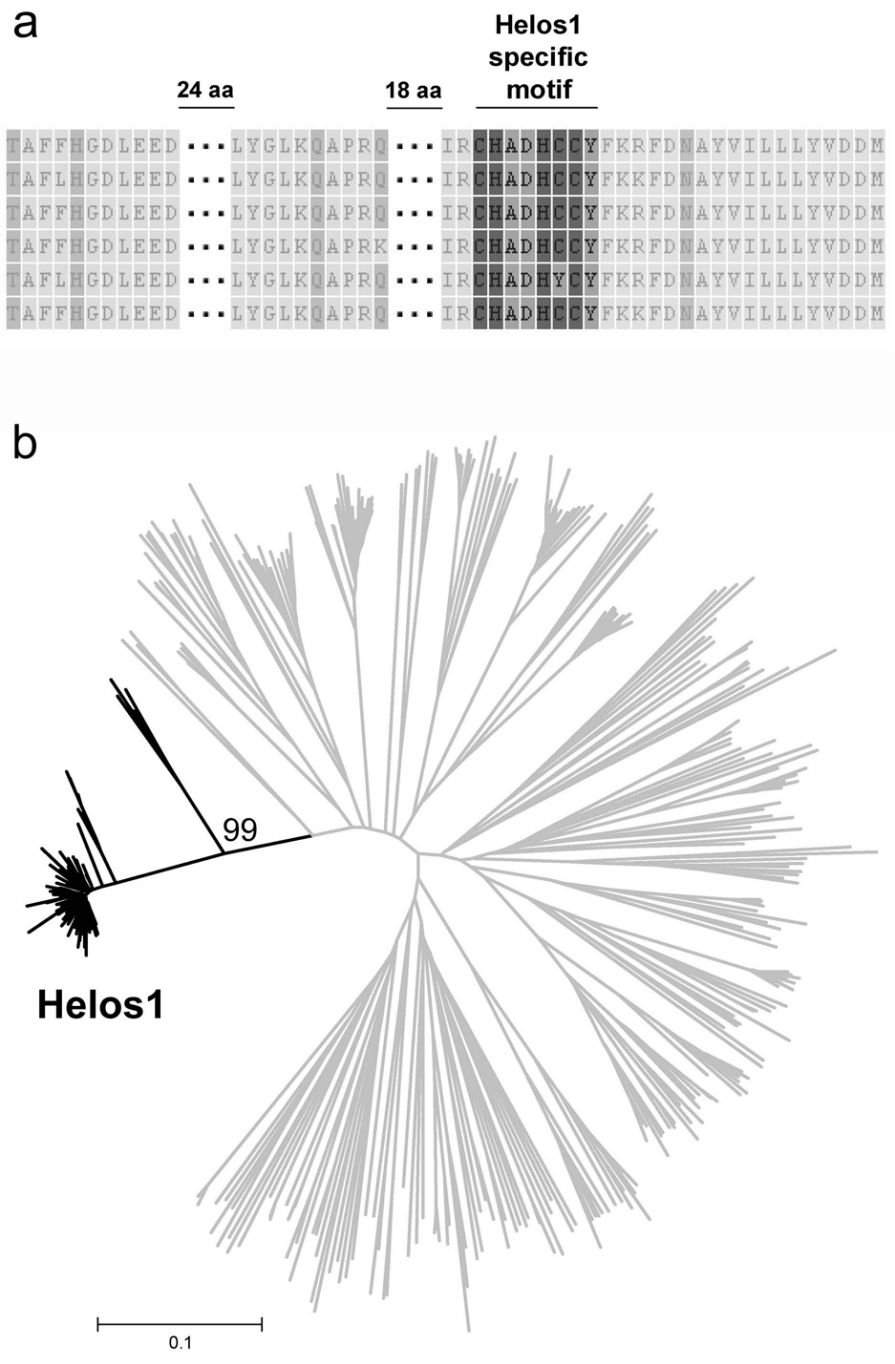
**Characteristics and phylogenetic position of Helos1**. **a **- Helos1 sequences aligned on the amino acid level. The highly conserved Helos1-specific motif is highlighted. **b **- Neighbor-joining phylogenetic tree of Ty1-*copia*. Branches leading to Helos1 sequences are depicted in black. A bootstrap value supporting the Helos1 clade is shown above the branch. The scale bar indicates Tamura 3-parameter distance.

Our observations suggest that the activities of the Ty1-*copia *superfamily have a causal relationship with the C_/n_-value variation observed in the genus *Eleocharis*. The obtained Ty1-*copia *sequences (Additional file 6) were analyzed for the presence of putative predominant families, under the expectation that predominant families in host genomes will also be overrepresented in amplicons, resulting in a higher probability of being cloned and sequenced (see below) [40,41]. The largest identified Ty1-*copia *family accounted for 109 out of 422 (26%) sequences of high nucleotide similarity (average divergence of less than 6%) and shared highly specific amino acid motifs (Figure 2). Because we did not find any similarity to previously described Ty1-*copia *families, this family was named Helos1 [GenBank: GU976288-GU976396]. Helos1 was found in 15 out of 16 analyzed species (with the exception of *E. interstincta*), indicating an ancestral origin for Helos1. As a potential candidate involved in genome size variation in the genus *Eleocharis*, the density of Helos1 was also quantified. The highest densities of Helos1 were observed in species at the top of the tree (Figure 1), and Helos1 density was significantly correlated with C_/n _(Table 2). This pattern was also observed for Ty1-*copia *as a whole, suggesting that the higher densities of Ty1-*copia *found in evolutionarily younger species were caused, at least in part, by Helos1 activity (Figure 1; Table 2).

### Helos1 was recently transposed in species that diverged later in evolutionary time

Retrotransposons are epigenetically silenced when they are young (recently amplified) [16]. Deamination of methylated cytosines leads to higher rates of transitions to thymines and elevated transition:transversion (Ts:Tv) ratios. Hence, recently transposed families/subfamilies should exhibit higher Ts:Tv ratios [16,42]. Therefore, we calculated the Ts:Tv ratio for Helos1 in the analyzed species. We observed an increasing Ts:Tv ratio for Helos1 in recently diverged species, with the lowest value found in *E. quinqueflora *(approx. 1.2) and the highest value found in *E. mamillata *(approx. 4.1). We also detected a positive correlation between independent phylogenetic contrasts [43-45] of Ts:Tv ratio and Helos1 density (Pearson correlation R = 0.619, p < 0.05). This finding suggests that the higher density of Helos1 in more recently diverging species is due to its recent amplification.

## Conclusions

As indicated by the positive correlation between genome size and chromosome number, our results confirm previous assumptions [35] that polyploidy occurs in the genus *Eleocharis*. However, the small genome sizes and high chromosome counts observed in our study indicate that agmatoploidy is the most important process determining chromosome number in the subgenera *Limnochloa *and *Zinserlingia*.

To our knowledge, this is the first study addressing LTR retrotransposons in plants with holokinetic chromosomes. Our data strongly suggest that Ty1-*copia*/Helos1 played an important role in the evolution of both genome size and karyotype in the genus *Eleocharis*, especially in the type subseries. In addition, the occurrence of both polyploidy events and Ty1-*copia*/Helos1 amplification is correlated with genome size in the phylogeny of the genus *Eleocharis *(Table 2). This relationship might help to explain why the evolutionarily youngest clade, subseries *Eleocharis *(Figure 1), occurs almost exclusively in temperate and boreal climatic zones, whereas the majority of genus diversity is located in tropical and subtropical zones [46].

As transposable elements, LTR retrotransposons have the strong potential to generate genic and genomic variation on which natural selection can act to create new species [12,47]. Although large genome rearrangements and/or TE insertions into genic regions are usually deleterious rather than beneficial, TE-induced mutations and alterations need not be adaptive to facilitate speciation. According to the epi-transposon hypothesis recently proposed by Zeh et al. [48], the non-adaptive impacts of TE activity can displace the population from its adaptive peak on the fitness landscape. Subsequently, a new peak is reached by natural selection, favoring beneficial non-transposon mutations [47,48]. Because species from the subseries *Eleocharis *are polyploid [[35,49-51], this study], they carry duplicate or multiple copies of all genes, and thus, the negative consequences of TE activity can be balanced [52,53]. A greater number of TEs within a genome increases the potential to produce fecund lineages with strong abilities to evolve and adapt to various conditions [47,48]. A relationship between the latitude and activity of the *mariner *transposable element has already been observed in populations of *Drosophila simulans *[54]. The authors of that study discussed the possibility that the invasion of new stressful habitats triggered the activity of *mariner*, which subsequently produced variation for natural selection. The occurrence of polyploidy has also been shown to be positively correlated with latitude in angiosperms [55-58]. It is possible that the widespread distribution and species richness of the subseries *Eleocharis *in temperate zones [46] was enhanced by transposable elements, which have larger operational genomic ranges for creating variation for natural selection and subsequent radiation in polyploids.

The above hypothesis remains to be tested. Full-length retrotransposons should be isolated and the position of their insertions within *Eleocharis *genomes should be determined. We believe that *Eleocharis *is a promising model for studying TE dynamics and genome evolution in a holokinetic system. The close taxonomical relationship between *Cyperaceae *and *Poaceae *could make these studies easier, as has been shown for the species *Luzula nivea *from the sister family *Juncaceae *[59].

## Methods

### Plant material

Samples were collected from wild populations and subsequently cultivated in water basins in an experimental garden of the Department of Botany and Zoology, Masaryk University, Brno. A list of sample locations is included in Additional file 7.

### Genome size measurements

Somatic DNA quantity (2C genome size) was estimated using flow cytometry (CyFlow SL; PARTEC GmbH) with the intercalating fluorochrome propidium iodide. A two-step procedure was performed (Otto 1 and 2; for more details, see reference [60]). Tomato (*Lycopersicon esculentum *'Stupické polní tyčkové rané'; 2C = 1.96 pg, [61]) and pea (*Pisum sativum *'Ctirad'; 2C = 9.09, [62]) were used as internal standards.

### DNA isolation

Plant tissue was ground into a fine powder in liquid nitrogen, and total genomic DNA was extracted using the DNeasy Plant Mini Kit (Qiagen) following the manufacturer's protocol. DNA concentrations were determined by measuring the absorbance of the samples at 260 nm.

### Amplification, cloning and sequencing

PCR was performed to amplify a conserved region of the *reverse transcriptase *gene using degenerate primers for Ty1-*copia *[37,38] and Ty3-*gypsy *[39], amplifying approximately 260-bp and 430-bp fragments, respectively. Amplifications were conducted in a final volume of 25 μl in the presence 50-200 ng of total genomic DNA, 1 μM of each primer, 80 μM of each nucleotide (dNTPs), 2 mM of MgCl_2 _and 2.5 U of PurpleTaq polymerase (TopBio) in the manufacturer's 1× PCR buffer. The following protocol was used to amplify both Ty1-*copia *and Ty3-*gypsy*: an initial denaturation step at 94°C for 2 min, followed by 40 cycles of 94°C for 30 s, 44°C for 1 min and 72°C for 30 s and a final elongation step at 72°C for 5 min.

The approximately 900-bp-long exon 8 of the *ppc *gene was amplified with the primer pair *ppc*-1465F (5'-TTTGGTCTCTCTYTTGTGCGTC-3') and *ppc*-2338R (5'-GRCGGAAATACTCAACAAAGCG-3'). ITS sequences of approximately 700 bp were amplified with the primer pair ITS1-UBZ (5'-GAACCTGCGGAAGGATCATTG-3') and ITS4-UBZ (5'-CCGCTTATTGATATGCTTAAACTC-3'). The cycling conditions were the same for both ITS and *ppc*, with an initial denaturation step at 94°C for 2 min, followed by 40 cycles of 94°C for 30 s, 56°C for 30 s and 72°C for 30 s and a final elongation step at 72°C for 5 min. Amplicons were run on a 1.2% agarose gel to verify their lengths. PCR products were cloned into pCR^®^2.1 vectors and transformed into TOP10F' competent cells (Invitrogen) according to the manufacturer's instructions. Selection of colonies for TEs was conducted as described previously [63,64]. Inserts of the desired length were evaluated using PCR with T7 and M13 primers. Sequencing was conducted by Macrogen, Inc.

### Sequence analysis

Sequences were aligned using the ClustalW algorithm, as implemented in the MEGA 4.0 software package [65]. Coding sequences of the *reverse transcriptase *and *phosphoenolpyruvate carboxylase *(*ppc*) genes were aligned at the amino acid level. If the open reading frame was destroyed due to indels, gaps were inserted to restore it. ITS sequences were aligned at the nucleotide level. All sequences were run through BLAST to check their homologies to retroelements, ITS and the *ppc *genes. Consensus sequences for ITS and *ppc *for each species were prepared in BioEdit v7.0.9.0 [66]. The original sequences used for consensus construction are available on GenBank: ITS [GenBank: GU977050-GU977108] and *ppc *[GenBank: GU977109-GU977187]. Neighbor-joining phylogenetic tree of the analyzed species, based on concatenated alignments of ITS and *ppc *sequences, were constructed in MEGA 4.0 from nucleotide sequences using the Tamura 3-parameter model and pairwise deletion option with 1000 bootstrap replicates. The phylogeny of Ty1-*copia *and Ty3-*gypsy reverse transcriptase sequences *was inferred in the same way. MEGA 4.0 was also used to compute the Ts:Tv ratios of Helos1.

### Real-time PCR

Real-time PCR was performed using a Rotor-Gene 6000 thermocycler (Corbett Life Sciences). The primers for Ty1-*copia *and Ty3-*gypsy *were the same as those described above. New primers were designed for Helos1 and used to amplify the 144-bp fragment of *reverse transcriptase*: Helos1-F (5'-ATGTAGAGGCTGGTAAGGA-3'), corresponding to amino acid motif VEAGK, and Helos1-R (5'-ARCAGTGATCTGCATGAC-3'), corresponding to amino acid motif HADHC. Purified recombinant plasmids containing these *reverse transcriptase *fragments were used as external standards. Because we used degenerate primers, plasmids carrying *reverse transcriptase *sequences with different primer binding sites were mixed together. The mix was linearized with the BamHI restriction enzyme before being used for real-time PCR analysis. A 10-fold serial dilution of the stock solution of plasmid DNA was prepared to construct standard curves for absolute quantification. Samples were diluted to a final concentration of 3.5 ng/μl. The 20-μl reaction mixture contained 2 μl of plasmid DNA or diluted genomic DNA from the samples, 1 μM of each primer, 100 μM dNTPs, 3 mM of MgCl_2_, 1× EvaGreen intercalating dye and 1 U of CombiTaq polymerase (TopBio) in 1× PCR buffer. All standards and samples were measured in duplicate. The cycling conditions for Ty1-*copia *and Ty3-*gypsy *were the same as those described above. The cycling conditions used for Helos1 included an initial denaturation step at 94°C for 3 min, 40 cycles of 94°C for 30 s, 54°C for 30 s and 72°C for 30 s and a final elongation step at 72°C for 5 min. Fluorescence measurements were conducted at the end of each elongation step. A standard curve for Ty1-*copia *quantification was generated, covering four orders of magnitude, from 6.94 × 10^5 ^to 6.94 × 10^8 ^copies per reaction tube. The coefficient R^2 ^and reaction efficiency (E) were estimated to be 1.0 and 82%, respectively. The standard curve for the Helos1 analysis ranged over six orders of magnitude, from 7.08 × 10^3 ^to 7.08 × 10^8 ^copies per reaction tube, with R^2 ^> 0.999 and E = 89%. The standard curve for Ty3-*gypsy *quantification covered five orders of magnitude, from 1.35 × 10^4 ^to 1.35 × 10^8 ^copies per reaction tube, with R^2 ^> 0.999 and E = 89%. Quantification was followed by a melt curve analysis to verify the reaction specificity. In all cases, a single peak was observed, indicating a single product.

### Dot blot hybridization

For the quantification of Ty1-*copia *and Helos1, genomic DNA from each analyzed species was diluted to 100, 50, 25, 12.5 and 6.25 ng, except *E. acicularis *and *E. ovata*, which were diluted to 200, 100, 50, 25 and 12.5 ng. In the case of Ty3-*gypsy *quantification, all samples were diluted to 100, 50, 25, 12.5 and 6.25 ng. PCR products prepared from the same plasmid mixes that were used in real-time PCR quantification were used as standards. These standards were diluted as follows: Ty1-*copia*: 3-fold dilution series from 3.27 × 10^10 ^to 1.35 × 10^8 ^copies per dot; Ty3-*gypsy*: 4-fold dilution series from 5.41 × 10^9 ^to 2.11 × 10^7 ^copies per dot; Helos1: 3-fold dilution series from 3.33 × 10^9 ^to 4.11 × 10^7 ^copies per dot. Except for *E. cellulosa*, all samples and standards were measured in duplicate. PCR products were used as probes, which were labeled with digoxigenin-dUTP according to the manufacturer's instructions (DIG High Prime Labeling Kit, Roche). The genomic DNA samples and PCR products were denatured for 5 minutes at 98°C and subsequently immobilized on positively charged nylon membranes. Hybridization was performed using a DIG hybridization kit (Roche). The hybridization temperature was set to maintain 80-100% homology. Hybridization procedures, washing and detection were carried out according to the manufacturer's instructions. Integrated densities used for the calculation of copy numbers were obtained using ImageJ software.

### Independent phylogenetic contrasts

The presence of a phylogenetic signal and correlations between studied traits were tested based on the comparison of phylogenetically independent contrasts [43,44]. Independent contrasts were calculated using the Analysis of Traits (AOT module) by David Ackerly, included in the Phylocom 4.1 package [45]. All tested traits were considered to be continuous in the analyses. The original branch lengths of the phylogenetic tree were log-transformed prior to all analyses. The significance of correlation coefficients was obtained using the table of critical values for the Pearson correlation. Tests of the phylogenetic signals were based on 999 randomizations.

## Authors' contributions

FZ carried out DNA isolations, PCR amplifications, and sequence analyses and participated in the cloning, real-time PCR, dot blots, statistical analyses, genome size measurement, design of the study and drafting the manuscript. JŠ participated in the cloning, real-time PCR quantification, dot blots and design of the study. PŠ participated in the design of the study and statistical analyses. PB conceived of the study, participated in the genome size measurement, and the design and coordination, and helped to draft the manuscript. All authors read and approved the final manuscript.

## Supplementary Material

Additional file 1 **Concatenated alignment of consensus sequences for ITS and *ppc*. **The alignment used for the construction of the phylogenetic tree for the analyzed species. Sequence identification: copEmam13-cop (Ty1-*copia*), E (Eleocharis), mam (first three letters of species name), 13 (number of clone). Click here for file

Additional file 2 **Alignment of Ty1-*copia *sequences**. The alignment used for the construction of the phylogenetic tree of Ty1-*copia *sequences. Sequence identification: copEmam13-cop (Ty1-*copia*), E (Eleocharis), mam (first three letters of species name), 13 (number of clone). Click here for file

Additional file 3 **Alignment of Ty3-*gypsy *sequences**. The alignment used for the construction of the phylogenetic tree of Ty3-*gypsy *sequences. Sequence identification: gypEaus27-gyp (Ty1-*gypsy*), E (Eleocharis), aus (first three letters of species name), 27 (number of clone). Click here for file

Additional file 4 Number of sequenced LTR retrotransposon clones per species and their respective GenBank accession numbers Click here for file

Additional file 5 **Neighbor-joining phylogenetic tree of Ty3-*gypsy***. Species-specific clades of *Eleocharis ovata*, *E. intersticta*, *E. cellulosa *and *E. quinqueflora *are highlighted and depicted in blue. Only bootstrap values higher than 50 are shown below branches. Sequence identification: gypEaus27-gyp (Ty1-*gypsy*), E (Eleocharis), aus (first three letters of species name), 27 (number of clone). Click here for file

Additional file 6 **Neighbor-joining phylogenetic tree of Ty1-*copia*. **Sequences corresponding to Helos1 are highlighted and depicted in red. Only bootstrap values higher than 50 are shown above branches. Sequence identification: copEmam13-cop (Ty1-*copia*), E (Eleocharis), mam (first three letters of species name), 13 (number of clone). Click here for file

Additional file 7 **List of plant samples**. The list includes full taxonomic names of analyzed species and their locations, including GPS positions, names of collectors and dates of collections. Click here for file

## References

[B1] Wicker T Sabot F Hua-Van A Bennetzen JL Capy P Chalhoub B Flavell A Leroy P Morgante M Panaud O A unified classification system for eukaryotic transposable elements Nature Reviews Genetics 2007 8 12 973 982 10.1038/nrg2165 17984973

[B2] Kumar A Bennetzen JL Plant retrotransposons Annual Review of Genetics 1999 33 479 532 10.1146/annurev.genet.33.1.479 10690416

[B3] Kumar A Bennetzen J Retrotransposons: central players in the structure, evolution and function of plant genomes Trends in plant science 2000 5 12 509 510 10.1016/S1360-1385(00)01760-X 11200422

[B4] Boeke JD Corces VG Transcription and reverse transcription of retrotransposons Annual Review of Microbiology 1989 43 403 434 10.1146/annurev.mi.43.100189.002155 2552899

[B5] Wilhelm M Wilhelm FX Reverse transcription of retroviruses and LTR retrotransposons Cellular and Molecular Life Sciences 2001 58 9 1246 1262 10.1007/PL00000937 11577982 PMC11337404

[B6] Kashkush K Feldman M Levy AA Transcriptional activation of retrotransposons alters the expression of adjacent genes in wheat Nature Genetics 2003 33 1 102 106 10.1038/ng1063 12483211

[B7] Hollister JD Gaut BS Epigenetic silencing of transposable elements: A trade-off between reduced transposition and deleterious effects on neighboring gene expression Genome Research 2009 19 8 1419 1428 10.1101/gr.091678.109 19478138 PMC2720190

[B8] Lockton S Gaut BS The Contribution of Transposable Elements to Expressed Coding Sequence in Arabidopsis thaliana Journal of Molecular Evolution 2009 68 1 80 89 10.1007/s00239-008-9190-5 19125217

[B9] Lonnig WE Saedler H Chromosome rearrangements and transposable elements Annual Review of Genetics 2002 36 389 410 10.1146/annurev.genet.36.040202.092802 12429698

[B10] Vitte C Panaud O LTR retrotransposons and flowering plant genome size: emergence of the increase/decrease model Cytogenetic and Genome Research 2005 110 1-4 91 107 10.1159/000084941 16093661

[B11] Grandbastien M Audeon C Bonnivard E Casacuberta JM Chalhoub B Costa APP Le QH Melayah D Petit M Poncet C Stress activation and genomic impact of Tnt1 retrotransposons in Solanaceae Cytogenetic and Genome Research 2005 110 1-4 229 241 10.1159/000084957 16093677

[B12] Mansour A Epigenetic activation of genomic retrotransposons Journal of Cell and Molecular Biology 2007 6 2 99 107

[B13] Okamoto H Hirochika H Silencing of transposable elements in plants Trends in Plant Science 2001 6 11 527 534 10.1016/S1360-1385(01)02105-7 11701381

[B14] Matsuoka Y Tsunewaki K Evolutionary dynamics of Ty1-copia group retrotransposons in grass shown by reverse transcriptase domain analysis Molecular Biology and Evolution 1999 16 2 208 217 10028288 10.1093/oxfordjournals.molbev.a026103

[B15] Navarro-Quezada A Schoen DJ Sequence evolution and copy number of Ty1-copia retrotransposons in diverse plant genomes Proceedings of the National Academy of Sciences of the United States of America 2002 99 1 268 273 10.1073/pnas.012422299 11752395 PMC117550

[B16] Baucom RS Estill JC Leebens-Mack J Bennetzen JL Natural selection on gene function drives the evolution of LTR retrotransposon families in the rice genome Genome Research 2009 19 2 243 254 10.1101/gr.083360.108 19029538 PMC2652206

[B17] Capy P Gasperi G Biemont C Bazin C Stress and transposable elements: co-evolution or useful parasites? Heredity 2000 85 2 101 106 10.1046/j.1365-2540.2000.00751.x 11012710

[B18] Comai L Tyagi AP Winter K Holmes-Davis R Reynolds SH Stevens Y Byers B Phenotypic instability and rapid gene silencing in newly formed arabidopsis allotetraploids Plant Cell 2000 12 9 1551 1567 10.1105/tpc.12.9.1551 11006331 PMC149069

[B19] Baumel A Ainouche M Kalendar R Schulman AH Retrotransposons and genomic stability in populations of the young allopolyploid species Spartina anglica CE Hubbard (Poaceae) Molecular Biology and Evolution 2002 19 8 1218 1227 12140233 10.1093/oxfordjournals.molbev.a004182

[B20] Parisod C Alix K Just J Petit M Sarilar V Mhiri C Ainouche M Chalhoub B Grandbastien MA Impact of transposable elements on the organization and function of allopolyploid genomes New Phytologist 2010 186 1 37 45 10.1111/j.1469-8137.2009.03096.x 20002321

[B21] Piegu B Guyot R Picault N Roulin A Saniyal A Kim H Collura K Brar DS Jackson S Wing RA Doubling genome size without polyploidization: Dynamics of retrotransposition-driven genomic expansions in Oryza australiensis, a wild relative of rice Genome Research 2006 16 10 1262 1269 10.1101/gr.5290206 16963705 PMC1581435

[B22] Soleimani VD Baum BR Johnson DA Quantification of the retrotransposon BARE-1 reveals the dynamic nature of the barley genome Genome 2006 49 4 389 396 10.1139/G05-119 16699559

[B23] Kalendar R Tanskanen J Immonen S Nevo E Schulman AH Genome evolution of wild barley (Hordeum spontaneum) by BARE-1 retrotransposon dynamics in response to sharp microclimatic divergence Proceedings of the National Academy of Sciences of the United States of America 2000 97 12 6603 6607 10.1073/pnas.110587497 10823912 PMC18673

[B24] Wendel JF Wessler SR Retrotransposon-mediated genome evolution on a local ecological scale Proceedings of the National Academy of Sciences of the United States of America 2000 97 12 6250 6252 10.1073/pnas.97.12.6250 10841529 PMC33996

[B25] Bennett MD Leitch LI Plant DNA *C*-values database (release 4.0, Oct. 2005) 2004 http://www.kew.org/cvalues/

[B26] Roalson EH Hinchliff CE Trevisan R da Silva CRM Phylogenetic Relationships in Eleocharis (Cyperaceae): C-4 Photosynthesis Origins and Patterns of Diversification in the Spikerushes Systematic Botany 2010 35 2 257 271 10.1600/036364410791638270

[B27] Blomberg SP Garland T Ives AR Testing for phylogenetic signal in comparative data: Behavioral traits are more labile Evolution 2003 57 4 717 745 12778543 10.1111/j.0014-3820.2003.tb00285.x

[B28] Roalson EH Friar EA Infrageneric classification of Eleocharis (Cyperaceae) revisited: Evidence from the internal transcribed spacer (ITS) region of nuclear ribosomal DNA Systematic Botany 2000 25 2 323 336 10.2307/2666645

[B29] Yano O Katsuyama T Tsubota H Hoshino T Molecular phylogeny of Japanese Eleocharis (Cyperaceae) based on ITS sequence data, and chromosomal evolution Journal of Plant Research 2004 117 5 409 419 10.1007/s10265-004-0173-3 15372307

[B30] Preston ML Pearman DA Dines TD New atlas of the British & Irish Flora 2002 New York: Oxford Univ. Press

[B31] Nishikawa K Furuta Y Ishitobi K Chromosomal evolution in genus Carex as viewed from nuclear-dna content, with special reference to its aneuploidy Japanese Journal of Genetics 1984 59 5 465 472 10.1266/jjg.59.465

[B32] Hipp AL Rothrock PE Roalson EH The Evolution of Chromosome Arrangements in Carex (Cyperaceae) Botanical Review 2009 75 1 96 109 10.1007/s12229-008-9022-8

[B33] Roalson EH McCubbin GA Whitkus R Chromosome evolution in Cyperales 2007 23 Claremont, CA, ETATS-UNIS: Rancho Santa Ana Botanic Garden

[B34] Roalson EH A synopsis of chromosome number variation in the Cyperaceae Botanical Review 2008 74 2 209 393 10.1007/s12229-008-9011-y

[B35] Da Silva CRM Trevisan R González-Elizondo MS Ferreira JM Vanzela ALL Karyotypic diversification and its contribution to the taxonomy of Eleocharis (Cyperaceae) from Brazil Australian Journal of Botany 2010 58 1 49 60 10.1071/BT09185

[B36] Smarda P Bures P Horova L Foggi B Rossi G Genome size and GC content evolution of Festuca: Ancestral expansion and subsequent reduction Annals of Botany 2008 101 3 421 433 10.1093/aob/mcm307 18158307 PMC2701825

[B37] Flavell AJ Dunbar E Anderson R Pearce SR Hartley R Kumar A Ty1-copia group retrotransposons are ubiquitous and heterogeneous in higher-plants Nucleic Acids Research 1992 20 14 3639 3644 10.1093/nar/20.14.3639 1379359 PMC334012

[B38] Flavell AJ Smith DB Kumar A Extreme heterogeneity of Ty1-copia group retrotransposons in plants Molecular &amp; General Genetics 1992 231 2 233 242 10.1007/BF00279796 1370976

[B39] Kumekawa N Ohtsubo E Ohtsubo H Identification and phylogenetic analysis of gypsy-type retrotransposons in the plant kingdom Genes &amp; Genetic Systems 1999 74 6 299 307 10.1266/ggs.74.299 10791026

[B40] Park JM Schneeweiss GM Weiss-Schneeweiss H Diversity and evolution of Ty1-copia and Ty3-gypsy retroelements in the non-photo synthetic flowering plants Orobanche and Phelipanche (Orobanchaceae) Gene 2007 387 1-2 75 86 10.1016/j.gene.2006.08.012 17008031

[B41] Ungerer MC Strakosh SC Stimpson KM Proliferation of Ty3/gypsy-like retrotransposons in hybrid sunflower taxa inferred from phylogenetic data Bmc Biology 2009 7 10.1186/1741-7007-7-40 19594956 PMC2715380

[B42] Whitford R Baumann U Sutton T Gumaelius L Wolters P Tingey S Able JA Langridge P Identification of transposons, retroelements, and a gene family predominantly expressed in floral tissues in chromosome 3DS of the hexaploid wheat progenitor Aegilops tauschii Functional &amp; Integrative Genomics 2007 7 1 37 52 10.1007/s10142-006-0026-3 16534632

[B43] Felsenstein J Phylogenies and the comparative method American Naturalist 1985 125 1 1 15 10.1086/284325 31094602

[B44] Garland T Harvey PH Ives AR Procedures for the analysis of comparative data using phylogenetically independent contrasts Systematic Biology 1992 41 1 18 32

[B45] Webb CO Ackerly DD Kembel SW Phylocom: software for the analysis of phylogenetic community structure and trait evolution Bioinformatics 2008 24 18 2098 2100 10.1093/bioinformatics/btn358 18678590

[B46] Svenson HK Monographic studies in the genus *Eleocharis *V Rhodora 1939 41 1 19 43-77, 90-110

[B47] Oliver KR Greene WK Transposable elements: powerful facilitators of evolution Bioessays 2009 31 7 703 714 10.1002/bies.200800219 19415638

[B48] Zeh DW Zeh JA Ishida Y Transposable elements and an epigenetic basis for punctuated equilibria Bioessays 2009 31 7 715 726 10.1002/bies.200900026 19472370

[B49] Bures P A high polyploid Eleocharis uniglumis sl (Cyperaceae) from Central and Southeastern Europe Folia Geobotanica 1998 33 4 429 439 10.1007/BF02803644

[B50] Bureš P Zedek F Šmarda P Rotreklová O Hrálová I Genome size in *Cyperaceae* The Comparative Biology of the Monocotyledons: 2008; Copenhagen, Denmark 2008 University of Copenhagen 13 14

[B51] da Silva CRM Gonzalez-Elizondo MS Rego L Torezan JMD Vanzela ALL Cytogenetical and cytotaxonomical analysis of some Brazilian species of Eleocharis (Cyperaceae) Australian Journal of Botany 2008 56 1 82 90 10.1071/BT07017

[B52] Matzke MA Matzke AJM Polyploidy and transposons Trends in Ecology &amp; Evolution 1998 13 6 241 241 10.1016/s0169-5347(98)01390-1 21238281

[B53] Matzke MA Scheid OM Matzke AJM Rapid structural and epigenetic changes in polyploid and aneuploid genomes Bioessays 1999 21 9 761 767 10.1002/(SICI)1521-1878(199909)21:9<761::AID-BIES7>3.0.CO;2-C 10462416

[B54] Picot S Wallau GL Loreto ELS Heredia FO Hua-Van A Capy P The mariner transposable element in natural populations of Drosophila simulans Heredity 2008 101 1 53 59 10.1038/hdy.2008.27 18461087

[B55] Löve A Löve D Arctic polyploidy Proc Genet Soc Can 1957 2 23 27

[B56] Stebbins GL Polyploidy and the distribution of the arctic-alpine flora-new evidence and a new approach Botanica Helvetica 1984 94 1 1 13

[B57] Brochmann C Brysting AK Alsos IG Borgen L Grundt HH Scheen AC Elven R Polyploidy in arctic plants Biological Journal of the Linnean Society 2004 82 4 521 536 10.1111/j.1095-8312.2004.00337.x

[B58] Guggisberg A Mansion G Conti E Disentangling Reticulate Evolution in an Arctic-Alpine Polyploid Complex Systematic Biology 2009 58 1 55 73 10.1093/sysbio/syp010 20525568

[B59] Haizel T Lim YK Leitch AR Moore G Molecular analysis of holocentric centromeres of Luzula species Cytogenetic and Genome Research 2005 109 1-3 134 143 10.1159/000082392 15753569

[B60] Bures P Wang YF Horova L Suda J Genome size variation in Central European species of Cirsium (Compositae) and their natural hybrids Annals of Botany 2004 94 3 353 363 10.1093/aob/mch151 15292040 PMC4242176

[B61] Dolezel J Greilhuber J Lucretti S Meister A Lysak MA Nardi L Obermayer R Plant genome size estimation by flow cytometry: Inter-laboratory comparison Annals of Botany 1998 82 17 26 10.1006/anbo.1998.0730

[B62] Dolezel J Sgorbati S Lucretti S Comparison of 3 DNA fluorochromes for flow cytometric estimation of nuclear-DNA content in plants Physiologia Plantarum 1992 85 4 625 631 10.1111/j.1399-3054.1992.tb04764.x

[B63] Park JM Schneeweiss GM Weiss-Schneeweiss H Diversity and evolution of Ty1-copia and Ty3-gypsy retroelements in the non-photo synthetic flowering plants Orobanche and Phelipanche (Orobanchaceae) Gene 2007 387 1-2 75 86 10.1016/j.gene.2006.08.012 17008031

[B64] Ruas CF Weiss-Schneeweiss H Stuessy TF Samuel MR Pedrosa-Harand A Tremetsberger K Ruas PM Schluter PM Herrera MAO Konig C Characterization, genomic organization and chromosomal distribution of Ty1-copia retrotransposons in species of Hypochaeris (Asteraceae) Gene 2008 412 1-2 39 49 10.1016/j.gene.2008.01.009 18302977

[B65] Tamura K Dudley J Nei M Kumar S MEGA4: Molecular evolutionary genetics analysis (MEGA) software version 4.0 Molecular Biology and Evolution 2007 24 8 1596 1599 10.1093/molbev/msm092 17488738

[B66] Hall TA BioEdit: a user-friendly biological sequence alignment editor and analysis program for Windows 95/98/NT Nucl Acids Symp Ser 1999 41 95 98

[B67] Gonzalez-Elizondo MS Peterson PM A classification of and key to the supraspecific taxa in Eleocharis (Cyperaceae) Taxon 1997 46 3 433 449 10.2307/1224386

[B68] Bures P Rotreklova O Stoneberg Holt SD Pikner R Cytogeographical survey of Eleocharis subser. Eleocharis in Europe-1: Eleocharis palustris Folia Geobotanica 2004 39 3 235 257 10.1007/BF02804780

[B69] Rotreklova O Bures P Grulich V Chromosome numbers for some species of vascular plants from Europe Biologia 2004 59 4 425 433

