# Supplementary figures and images for: Correlated evolution of LTR retrotransposons and genome size in the genus eleocharis
Source: BMC Plant Biol. 2010 Nov 30;10:265. doi: 10.1186/1471-2229-10-265 (PMC3095338; doi:10.1186/1471-2229-10-265)

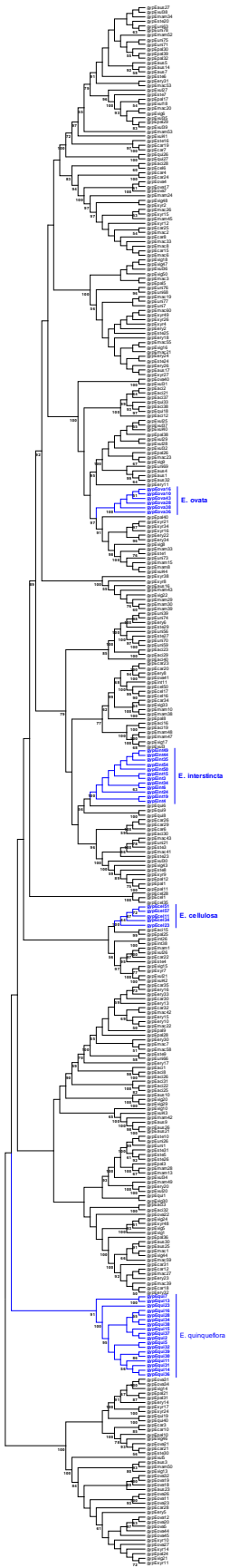

Supplement: Additional file 5 — Neighbor-joining phylogenetic tree of Ty3-gypsy. Species-specific clades of Eleocharis ovata, E. intersticta, E. cellulosa and E. quinqueflora are highlighted and depicted in blue. Only bootstrap values higher than 50 are shown below branches. Sequence identification: gypEaus27-gyp (Ty1-gypsy), E (Eleocharis), aus (first three letters of species name), 27 (number of clone). [file 1471-2229-10-265-S5.PDF]

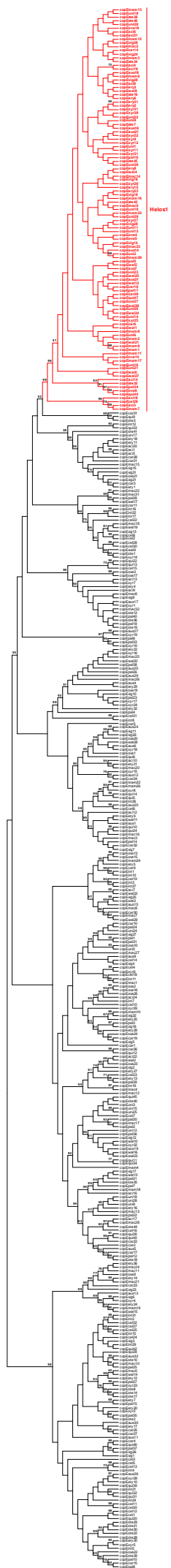

Supplement: Additional file 6 — Neighbor-joining phylogenetic tree of Ty1-copia. Sequences corresponding to Helos1 are highlighted and depicted in red. Only bootstrap values higher than 50 are shown above branches. Sequence identification: copEmam13-cop (Ty1-copia), E (Eleocharis), mam (first three letters of species name), 13 (number of clone). [file 1471-2229-10-265-S6.PDF]
