# Supplementary material for: Correlated evolution of LTR retrotransposons and genome size in the genus eleocharis
Source: BMC Plant Biol. 2010 Nov 30;10:265. doi: 10.1186/1471-2229-10-265 (PMC3095338; doi:10.1186/1471-2229-10-265)
Supplement: Additional file 7 — List of plant samples. The list includes full taxonomic names of analyzed species and their locations, including GPS positions, names of collectors and dates of collections. [file 1471-2229-10-265-S7.DOC]

**The list of sample locations:**

*E.* ***acicularis*** (L.) Roem. & Schult.: Czech Republic, Ořechov: Malý Chlostov Pond W of the village, 49°21'05.1'' N, 16°07'32.5'' E, coll. P. Bureš & J. Šmerda Sep/2006.

*E.* ***acicularis*** (L.) Roem. & Schult.: Czech Republic,Radostín: Velké Dářko Pond W of village 49°38'24.52"N, 15°54'22.932"E coll. P. Bureš & J. Šmerda Sep/2006

*E.* ***acicularis*** (L.) Roem. & Schult.: Czech Republic, Beskydy Horní Bečva dam S of village, leg P. Šmarda, Jun/2007 49°25'14.564"N, 18°19'4.418"E

*E.* ***acicularis*** (L.) Roem. & Schult.: Czech Republic, Břeclav, village of Lanžhot: meadow NW of forester's house Dúbravka 4 km SW of the village, coll. V. ŘEHOŘEK, Aug/1996. 48°41'41.823"N, 16°57'6.782"E.

*E.* ***acicularis*** (L.) Roem. & Schult.: Czech Republic, Světnov Hlinečník Pond W of the village coll. P. Bureš & J. Šmerda Sep/2006. 49°37'8.592"N, 15°56'51.842"E

*E.* ***acicularis*** (L.) Roem. & Schult.: Czech Republic, Hamry dam S of the village 49°43'16.271"N, 15°55'4.32"E coll. P. Bureš & J. Šmerda Sep/2006.

*E.* ***acicularis*** (L.) Roem. & Schult.: Czech Republic, Žďár nad Sázavou Vetla Pond S of the town 49°32'5.296"N, 15°56'44.978"Ecoll. P. Bureš & J. Šmerda Sep/2006.

*E. mamillata* subsp. ***austriaca*** (Hayek) Strandhede: Czech Republic, Jezerné (in Radhošťské Beskydy Mts.): pond Jezero N of the village, 49°23'31.6'' N, 18°16'33.0'' E, coll. P. Bureš & S. Sankar 10/Oct/2009.

*E.* ***cellulosa*** Torr.: Belize, Buena Vista: “Buena Vista” marsh, 1.5 km E from the village, 18°14'4'' N, 88°30'44'' W, coll. J. Košnar 2008.

*E.* ***erythropoda*** Steud. U. S. A. Montana, below Gene Reservoir, 47°53'17'' N, 107°14'11'' W, coll. R. A. Stoneberg & S. D. Stoneberg Holt 19/Sep/2001.

*E.* ***interstincta*** (Vahl) Roem. & Schult.: Belize, San Estevan: “Quiet” marsh: Orange Walk, 2 km NW from the village, 18°10'2'' N, 88°31'29'' W, coll. J. Košnar 2008.

*E.* ***macrostachya*** Britton in Small [variant b sensu Smith et al. 2002]: U. S. A. Montana, near portable bull corral, E of road, S of Glasgow on Pines road, 47°59'16'' N, 106°43'27'' W, coll. R. A. Stoneberg & S. D. Stoneberg Holt 19/Sep/2001.

*E. mamillata* (H. Lindb.) H. Lindb. subsp. ***mamillata***: Czech Republic, Žďár nad Sázavou: W shore of water reservoir Pilská nádrž N of the town, 49°35'58.0'' N, 15°55'21.2'' E, coll. P. Bureš & J. Šmerda Sep./2006

*E.* ***ovata*** (Roth) Roem. & Schult.: Czech Republic, Žďár nad Sázavou: W shore of water reservoir Pilská nádrž N of the town, 49°35'58.0'' N, 15°55'21.2'' E, coll. P. Bureš & J. Šmerda Sep./2006

*E. palustris* (L.) Roem. & Schult. subsp. ***palustris***: Hungary, Pér (distr. Györ): wetland between fields 1 km WNW of the village, 47°37'14.5'' N, 17°47'28.8'' E, coll. P. Bureš & R. Pikner 1/Jul/1998.

*E.* ***quinqueflora*** (Hartm.) O.Schwarz: Czech Republic, Hluboká: wet meadow 0.6 km N of the village, 49°39'55.7'' N, 15°51'07.7'' E, coll. P. Bureš & J. Bureš 6/Jun/2008.

*E. uniglumis* subsp. ***sterneri*** Strandhede: Hungary, Hidegség (National Park Fertö) margin of brackish pools 0.5 km N of the village, 47°37'57.4'' N, 16°44'41.4'' E, coll. P. Bureš 17/Jul/1997

*E. uniglumis* (Link) Schult. subsp. ***uniglumis***: Sweden, Slite (Gotland Island), shore of brackish lake WSW of the town, 57°41'39.6'' N, 18°47'44.2'' E, coll. P. Bureš & M. Burešová, 24/Jul/2001.

*E. palustris* var. ***vigens*** L. H. Bailey: U. S. A. Montana, S of Glasgow Willow Creek road near Ball place, 47°;53';10'' N+106°;54';12'' W; coll. R. A. Stoneberg & S. D. Stoneberg Holt 19/Sep/2001.

*E. palustris* subsp. ***waltersii*** Bureš & Danihelka [= *E. vulgaris* Á.Löve & D.Löve]: Denmark: Kliplev (county Jylland): Hostrup Sø Lake, 54°57'47.8'' N, 9°25'13.9'' E, coll. P. Bureš & I. Burešová 7/Aug/1994.

*E.* ***xyridiformis*** Fernald & Brackett: U. S. A. Montana, near portable bull corral, E of road, S of Glasgow on Pines road, 47°59'16'' N, 106°43'27'' W, coll. R. A. Stoneberg & S. D. Stoneberg Holt 19/Sep/2001.

**References:**

Smith SG, Bruhl JJ, González-Elizondo MS. & Menapace FJ. 2002. *Eleocharis*. – In: Flora of North America, 23: 60–120. Oxford Univ. Press, New York, Oxford.
